# Biochemical Characterization of Halotolerant *Bacillus safensis* PM22 and Its Potential to Enhance Growth of Maize under Salinity Stress

**DOI:** 10.3390/plants11131721

**Published:** 2022-06-29

**Authors:** Muhammad Atif Azeem, Fahim Hussain Shah, Abid Ullah, Kishwar Ali, David Aaron Jones, Muhammad Ezaz Hasan Khan, Azad Ashraf

**Affiliations:** 1 Department of Plant Sciences, Quaid-i-Azam University, Islamabad 45320, Pakistan; atifazeem321@gmail.com (M.A.A.); fahimhussain112233@gmail.com (F.H.S.); 2 Botany Department, University of Malakand, Chakdara 18800, Pakistan; abidqau101@gmail.com; 3 College of General Education, University of Doha for Science and Technology, Arab League Street, Doha P.O. Box 24449, Qatar; mdezazhasan.khan@udst.edu.qa; 4 College of Health Sciences, University of Doha for Science and Technology, Arab League Street, Doha P.O. Box 24449, Qatar; davidaaron.jones@udst.edu.qa; 5 College of Engineering, University of Doha for Science and Technology, Arab League Street, Doha P.O. Box 24449, Qatar; azad.ashraf@udst.edu.qa

**Keywords:** rhizosphere, abiotic stresses, salinity stress, plant–microbe interactions, maize plant, osmoprotectants, antioxidant enzymes

## Abstract

Salinity stress is one of the primary abiotic stresses limiting crop growth and yield. Plants respond to salinity stress with several morphophysiological, molecular, and biochemical mechanisms, however, these mechanisms need to be improved further to cope with salt stress effectively. In this regard, the use of plant growth-promoting (PGP) and halotolerant bacteria is thought to be very efficient for enhancing growth and salinity tolerance in plants. The current study aims to assess *Bacillus safensis* PM22 for its ability to promote plant growth and resistance to salt. The PM22 produced substantial amounts of exopolysaccharides, indole-3-acetic acid, siderophore, and 1-aminocyclopropane-1-carboxylic acid deaminase (ACC-deaminase) under saline conditions. Additionally, inoculation of the halotolerant bacteria PM22 reduced the severity of salinity stress in plants and increased root and shoot length at various salt concentrations (0, 180, 240, and 300 mM). Furthermore, PM22-inoculated plants showed markedly enhanced photosynthetic pigment, carotenoid, leaf relative water content, 2,2-diphenyl-1-picrylhydrazyl (DPPH) activity, salt tolerance index, total soluble sugar, total protein, and ascorbic acid contents compared to non-inoculated control maize plants. PM22 substantially increased antioxidant (enzymatic and non-enzymatic) activities in maize plants, including ascorbate peroxidase, peroxidase, superoxide dismutase, catalase, total flavonoid, and phenol levels. Maize plants inoculated with PM22 also exhibited a significant reduction in electrolyte leakage, hydrogen peroxide, malondialdehyde, glycine betaine, and proline contents compared to non-inoculated control plants. These physiological appearances were further validated by quantitative reverse transcription-polymerase chain reaction (qRT-PCR), which revealed the upregulation of expression in genes responsible for stress tolerance. In the current investigation, *Bacillus safensis* PM22 showed plant growth-promoting and salt tolerance attributes and can be utilized as a bio-inoculant to improve yield in salt stress affected areas.

## 1. Introduction

Salinity is one of the serious environmental issues affecting plant growth and development which leads to a decrease in plant yield [1]. In arid and semi-arid regions, increased soil salinity is a severe problem that reduces agricultural productivity. There are 831 million hectares of salt-affected land on this planet, including 397 million hectares (Mha) of saline soils and 434 Mha of sodic soils. In Pakistan, the salt-affected area is 4.5 Mha [2,3,4]. Salinity is increasing at a rate of over 10% per year, and by 2050, it is predicted that salinity would affect 50% of agricultural land [3,5]. In saline soils, sodium chloride (NaCl) is the most common salt component that affects two biological processes namely, photosynthesis and protein synthesis [6]. In addition, NaCl reduces plant growth, declines crop productivity, and may ultimately lead to the death of plants [7]. However, plants respond to salt stress on a variety of levels, including systemic, localized (such as leaves, reproductive organs, and roots), cellular, biochemical, and molecular levels [8]. In addition, plants also produce several kinds of compatible enzymes, solutes, hormones, and ions to regulate homeostasis [9]. However, these mechanisms are insufficient to alleviate salt stress beyond a certain limit. Recently, several technologies have been developed to mitigate the effects of salt stress on plants, however, plant growth-promoting microorganisms provide an environmentally friendly and cost-effective strategy in this regard [10].

Plant growth-promoting bacteria (PGPB) proficiently stimulate plant growth by improving nutrient acquisition, phosphate solubilization, nitrogen fixation, iron sequestration, and production of 1-aminocyclopropane-1-carboxylic acid (ACC deaminase) and indole-3-acetic acid (IAA) [11]. In addition, PGPB influences plants indirectly by the synthesis of various chemicals (siderophores, antibiotics, extracellular hydrolytic enzymes, etc.) that can inhibit and/or suppress pathogen propagation [12]. PGPBs belonging to the *Bacillus* genus are widely utilized and commercialized as biocontrol agents in agricultural settings [13]. Microorganisms that promote plant growth are also used to improve stress tolerance in many crops [14]. There are several PGPBs, including the *Bacillus* species, which are extremely salt-tolerant [15]. This halotolerant *Bacillus* plays the main role in successful salt tolerance in plants due to their potential to participate in a variety of processes that take place in plants, such as hydraulic conductance, osmolyte accumulation, Na^+^ sequestration, and the maintenance of photosynthetic activities [16].

*Zea mays* L. (maize) is the third major cereal crop after rice and wheat which can be grown on a range of soils and climates. It belongs to the family Poaceae and is moderately tolerant to salt stress [17,18]. In accordance with the salinity-induced growth reduction of the biphasic model [19], decreased growth in cereals, especially in wheat, is caused by osmotic stress in the first phase and ionic toxicity in the second phase. Fortmeier and Schubert (1995) validated a similar model for growth decrease induced by salinity in maize, however, ion toxicity and the accompanying growth decline may occur in a relatively small amount in maize during the first period [20,21]. The temperament of maize toward salinity stress is associated with the excessive deposit of sodium ions (Na^+^) in their leaves [20]. Salt levels higher than 0.25 M affect maize plants by causing reduced growth and strict wilting [22].

The aim of this research was to analyze how inoculating *Bacillus safensis* PM22 influences maize growth and photosynthetic pigments under normal circumstances and salt stress of 180, 240, and 300 mM. The generation of several antioxidants (SOD, POD, CAT, APX) and non-enzymatic antioxidants (flavonoids and phenol contents) was also assessed to understand the translocation of Na^+^ and K^+^ in salinity stress. Furthermore, the expression pattern of plant growth promoting genes in maize plants inoculated with or without PM22 was studied under salt stress.

## 2. Results

### 2.1. This. Growth Status of Bacillus safensis PM22

**Figure 1:**
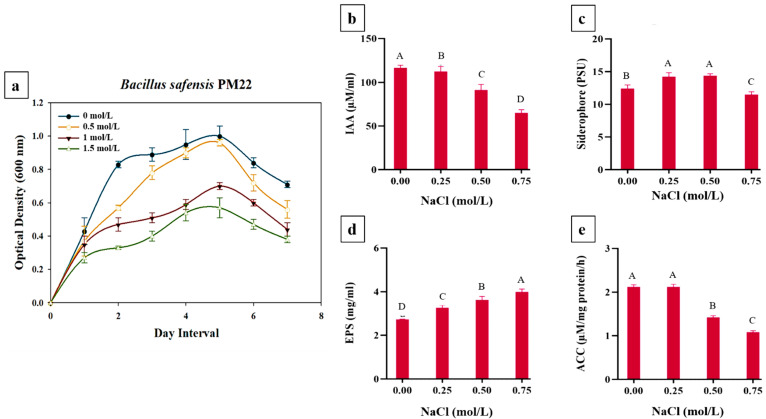
*B. safensis* PM22 growth curves at various salinity stress levels (0.0, 0.5, 1.0, and 1.5 mol/L) (**a**). Plant growth-promoting activities of *B. safensis* PM22 were investigated. IAA production (**b**), siderophore synthesis (**c**), EPS production (**d**), and ACC deaminase activity (**e**) were measured under various salt stress conditions. Means are used to summarize values, while bars represent standard errors. Tukey’s least significant difference (LSD) test used different alphabets to exhibit significantly different results (*p* = 0.05).

The resilience of *Bacillus safensis* PM22 to NaCl stress was tested by monitoring its growth using a spectrophotometer. PM22 tolerated 0.0, 0.5, 1.0, and 1.5 mol/L NaCl and displayed substantial growth at any concentrations provided (Figure 1a). In comparison to 1.0 and 1.5 mol/L sodium chloride concentrations, maximum growth was seen at 0.5 mol/L sodium chloride concentrations. On the fifth day of incubation, a growth curve analysis indicated the log phase. Control without stress had the maximum optical density.

### 2.2. Quantitative Evaluation of PGP Traits of the Selected Bacterial Strain

Plant growth-promoting traits were evaluated to observe the potential of *Bacillus safensis* PM22 under salt stress. Four different treatments (0.0, 0.25, 0.5, and 0.75 mol/L) were provided and each gave a different result against each PGP trait. IAA production was found to sustain up to 0.5 mol/L NaCl concentrations but reduced at 0.5 and 0.75 mol/L treatment (Figure 1b). Maximum production of IAA was noticed at 0.0 mmol/L NaCl concentration (116.52 ± 0.07). A similar trend was observed for ACC (2.19 ± 0.05) at 0.0 mol/L and reduced to 1.28 ± 0.05 at 0.75 mol/L (Figure 1e). An increase in siderophore was observed with the application of NaCl and the maximum value was recorded (14.39 ± 0.28) at 0.5 mol/L (Figure 1c). The level of ACC, IAA, and siderophore fell at salt treatment above 0.5 mol/L. Production of EPS was noticed to have a direct proportion with salt treatment. The maximum value of EPS (3.97.09 ± 0.13) was noted at 0.75 mol/L NaCl concentrations (Figure 1d).

### 2.3. Effects of Bacillus safensis PM22 on Maize Plant Growth under Salt Stress

Maize plants under various salinity conditions were used to assess the effectiveness of *Bacillus safensis* PM22 in reducing the effects of salt stress on plant growth. PM22-inoculated plants showed remarkable improvements in growth when compared to non-inoculated plants at all salt concentrations. In contrast to the control, inoculated maize plants showed a significant increase in shoot length (33.79%) specifically at 300 mM compared to non-inoculated control under the same levels of salt stress. Similarly, the highest rise in root length (26.94%) was found under 300 mM when compared to the non-inoculated respective control. In comparison with the control, inoculation with PM22 significantly increased the fresh weight of plants (*p* = 0.05). The maximum increase in fresh weight (37.22%) was noted at 300 mM in inoculated plants. The highest increase in dry weight (36.36%) and leaf area (28.13%) was observed at 300 and 240 mM NaCl, respectively, as compared to the control plants treated with respective salt doses (Table 1).

### 2.4. Pigments Content, Total Soluble Sugars, and Protein Contents

**Figure 2:**
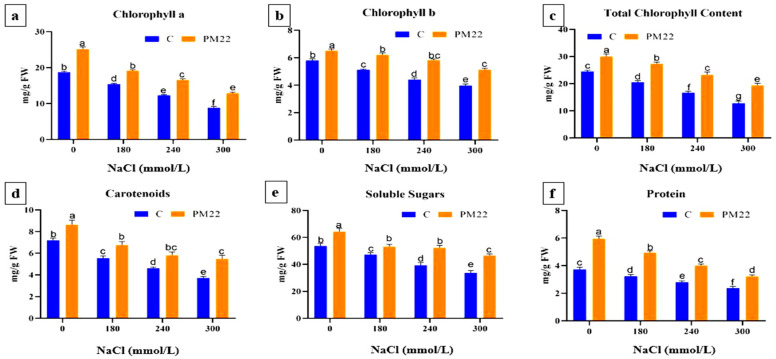
Impacts of various treatments on pigment and osmolyte contents of PM22-inoculated maize at different salt stress levels (0, 180, 240, and 300 mM). Soil containing non-inoculated seeds and soil with seeds inoculated with *B. Safensis* (PM22) were the two treatments. The values are the averages of three triplicates with standard errors of the mean. Different alphabets have significantly different values (*p* = 0.05) from each other, according to Tukey’s least significant difference test.

The effect of PM22 inoculation on the pigment content in maize was studied in both non-saline and saline conditions. PM22 inoculation markedly improved the chlorophyll in maize plants as compared to non-inoculated maize plants. The maximum enhancement in chlorophyll a was noted at 300 mM (31.67%) and 240 mM (25.86%) salinity stress in PM22-inoculated maize plants compared with the respective control (Figure 2a). The utmost increase in chlorophyll b was 43.46%, and 34.85% at 300 mM and 0 mM, respectively, in inoculated plants compared to controls (Figure 2b). Total chlorophyll contents were markedly improved at 300 mM (35.46%) followed by 240 mM (28.30%) salt stress for PM22-treated plants compared with their controls (Figure 2c). Similarly, carotenoid contents were increased in inoculated plants when compared with their non-inoculated control (Figure 2d).

Changes in osmoprotectants content were studied in maize plants under NaCl stress with and without inoculation with PM22. A significant upsurge in total soluble sugars (TSS) and protein contents was recorded in plants with the application of salt stress. The PM22-inoculated plants were exposed to a maximum rise in TSS and protein under control conditions (0 mM). In consideration of total soluble sugar contents, the maximum increase was observed at 240 mM (24.70%), then 300 mM (23.94%) NaCl when compared with the non-inoculated control (Figure 2e). Furthermore, protein contents were increased up to 37.41% at 300 mM NaCl stress as compared with non-inoculated plants (Figure 2f).

### 2.5. Relative Water Content and K^+^ and Na^+^ Content

**Figure 3:**
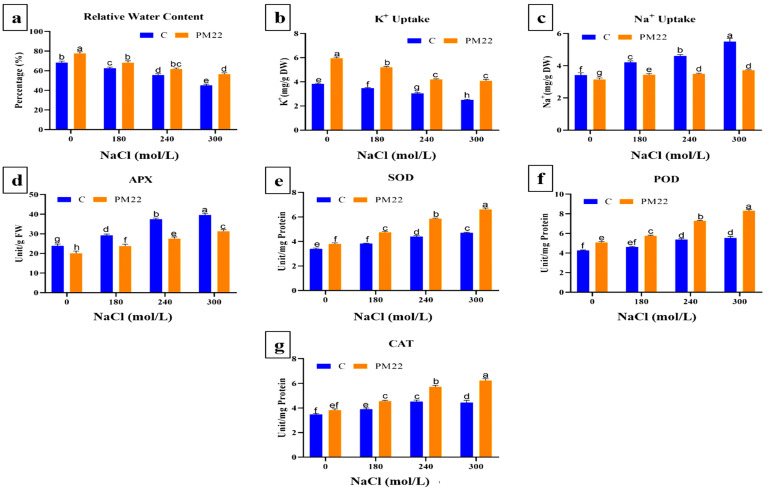
Impacts of various treatments on relative water content, ion homeostasis, and antioxidant activities of PM22-inoculated maize at different salt stress levels (0, 180, 240, and 300 mM). Soil containing non-inoculated seeds and soil with seeds inoculated with *B. Safensis* (PM22) were the two treatments. The values are the averages of three triplicates with standard errors of the mean. Different alphabets have significantly different values (*p* = 0.05) from each other, according to Tukey’s least significant difference test.

Salinity stress greatly affected the relative water content, potassium and sodium uptake. Maximum relative water content (RWC) was noted at 0 mM (20.37%) and 300 mM (14.55%) NaCl followed by 240 mM and 180 mM NaCl stress compared to maize plants without PM22 with applied salinity stress (Figure 3a). The elevation in Na^+^ accumulation in maize plants was noted with an increase in salt level from 0 to 300 mM (3.42–5.51 mg/g FW). However, the application of salt stress to PM22-inoculated plants showed a reduced amount of Na^+^ (7.89, 18.28, 24.07, 32.36%) compared with their respective controls (Figure 3c). Sodium chloride doses reduced K^+^ influx in maize plants. Though, PM22 application with different levels of NaCl treatments showed a significant increase in K^+^ accumulation (13.31, 23.04, 33.20, 35.95%) (Figure 3b).

### 2.6. Antioxidants Activities in Maize Plants Tissues

Antioxidant enzymes provide major support to plants by scavenging reactive oxygen species excessively produced in response to abiotic stresses. The antioxidant activities (APX, CAT, SOD, and POD) were assessed in PM22-inoculated and non-inoculated plants. An increase in the level of antioxidants was detected in plants with NaCl application and in plants inoculated with PM22 under salinity stress. The SOD activity was noted to be enhanced in inoculated plants under salinity stress. Inoculation of PM22 remarkably enhanced SOD activity by 10.76%, 19.53%, 24.87%, and 28.85% in 0, 180, 240 and 300 mM treatment, respectively, compared to non-inoculated plants (Figure 3e). At 240 and 300 mM of salt stress, PM22 significantly increased SOD content as compared to all studied treatments. The activity of POD in inoculated plants was meaningfully enhanced by 14.83, 20.13, 25.27, and 29.80% at 0 to 300 mM NaCl as compared to non-inoculated plants (Figure 3f). An increase in CAT activity by 9.37, 14.44, 20.52, and 28.38% was observed in inoculated plants compared with non-inoculated plants under 0, 180, 240, and 300 mM salt stress, respectively (Figure 3g). However, the APX value decreased by 10.58, 20.59, and 23% but at 300 mM treatment an 18.92% decrease was noted as compared with respective controls (Figure 3d). In comparison to other treatments, enzyme levels were considerably greater at higher levels of salt stress.

### 2.7. Proline, Glycine betaine, and Free Amino Acids Contents

**Figure 4:**
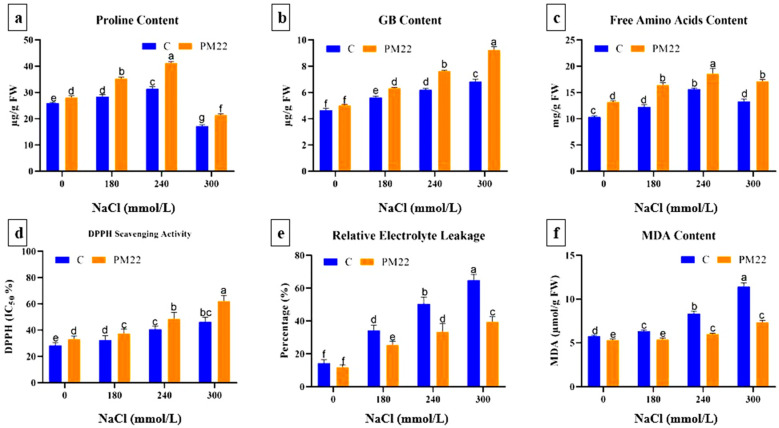
Impacts of various treatments on proline, glycine betaine, free amino acid contents, and oxidative stress indicators of PM22-inoculated maize at different salt stress levels (0, 180, 240, and 300 mM). Soil containing non-inoculated seeds and soil with seeds inoculated with *B. Safensis* (PM22) were the two treatments. The values are the averages of three triplicates with standard errors of the mean. Different alphabets have significantly different values (*p* = 0.05) from each other, according to Tukey’s least significant difference test.

Proline is known for its roles as an osmoprotectant, ROS scavenger, and stabilizer for subcellular structures of plant cells. GB also inhibits the generation of reactive oxygen species (ROS), safeguards photosynthetic apparatus, and increases the expression of stress-response genes. Plant proline and betaine contents were also measured to investigate the amounts of cell osmotic adjustment in shoots induced by salt. Although, as salt stress rose, the proline level of the plants increased. Results revealed that proline contents improved by 20.34%, 31.72%, and 38.37% (180, 240, and 300 mM salt, respectively) with the application of NaCl compared with the control (Figure 4a). *Bacillus safensis* PM22 inoculation increased the proline contents by 24.35%, 31.09%, and 37.01% in plants at 180, 240, and 300 mM NaCl salinity stress, respectively. As a sign of plant abiotic stress tolerance, concentrations of betaine were higher when the plants were under salt stress. The GB contents were increased by 20.66%, 34.16%, and 45.32% (180, 240, and 300 mM salt, respectively) with the application of NaCl compared with their non-inoculated control. *Bacillus safensis* PM22 inoculation increased the GB contents by 11.35%, 18.84%, and 22.73% in plants at 180, 240, and 300 mM NaCl stress, respectively (Figure 4b). Therefore, the application of *Bacillus safensis* PM22 ascertained a significant role in controlling betaine concentration in inoculated maize plants. Amino acids enhance the resilience of plants to abiotic stresses by promoting the metabolic machinery of plant cells. Amino acid metabolism in maize plants was changed when they were exposed to salt stress. In comparison to non-inoculated controls, free amino acids increased in PM22-treated plants at 300 mM (34.23%) and 240 mM (27.49%) salt concentrations (Figure 4c). The radical scavenging capacity of the maize plant was noted to be higher in plants inoculated with PM22 (Figure 4d).

### 2.8. Oxidative Stress Indicators

**Figure 5:**
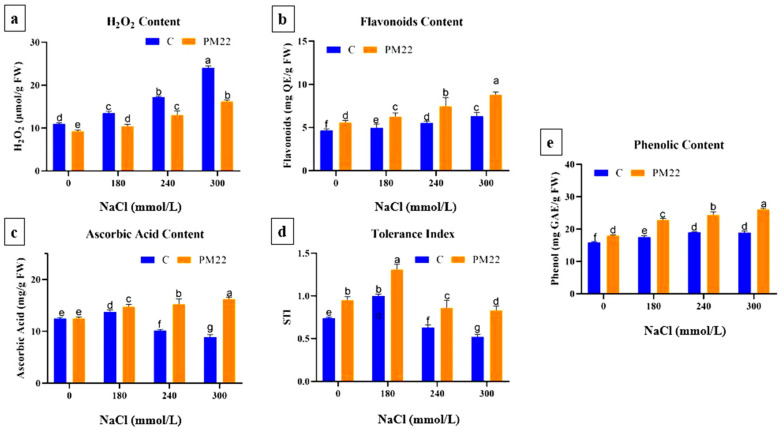
Impacts of various treatments on flavonoids, phenol, ascorbic acid contents, and salt tolerance index of PM22-inoculated maize at different salt stress levels (0, 180, 240, and 300 mmol/L). Soil containing non-inoculated seeds and soil with seeds inoculated with *B. Safensis* (PM22) were the two treatments. The values are the averages of three triplicates with standard errors of the mean. Different alphabets have significantly different values (*p* = 0.05) from each other, according to Tukey’s least significant difference test.

The status of oxidative stress was evaluated by estimating oxidative stress indicators. Untreated maize plants and those treated with *Bacillus safensis* PM22 were tested for the generation of oxidative stress biomarkers during salt stress. It was observed that by increasing the concentration of salt from zero to 300 mM, ELL, MDA, and H_2_O_2_ content also increased. *Bacillus safensis* PM22 inoculation treatments caused a noteworthy reduction in all traits when different concentrations of NaCl were applied (Figure 5). The maximum decrease in ELL (39.03%), MDA (44.26%) (Figure 4e,f), and H_2_O_2_ (44.83%) (Figure 5a) was marked in maize plants under 300 mM salinity stress compared to non-inoculated controls.

### 2.9. Flavonoids, Phenol, Ascorbic Acid Contents, and Salt Tolerance Index

Antioxidant compounds are effective in maintaining equilibrium between ROS production and the antioxidant system’s radical scavenging activity that ultimately leads to ROS detoxification in plants. Substantial decreases in flavonoids, phenol, and ascorbic acid contents were recorded in plants under salt stress. Plants inoculated with PM22 showed increased flavonoids, phenol, and ascorbic acid contents as compared with their respective controls under salt stress. Higher flavonoid content was observed at 300 mM (29.22%) and 240 mM (25.93%) NaCl than 180 mM NaCl stress as compared to non-inoculated control (Figure 5b). In consideration of phenol content, maximum increase occurred at 300 mM (40.41%) NaCl when compared with 0 mM (18.94%) (Figure 5e).

**Figure 6:**
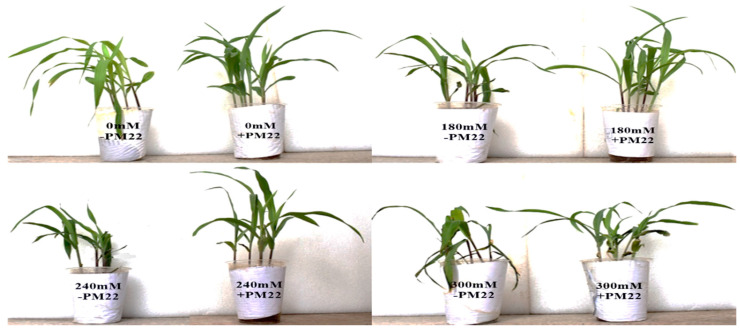
Effects of *Bacillus safensis* PM22 on the growth of maize under salinity stress.

Salt increment causes the plants to experience a decrease in ascorbic acid. However, the ascorbic acid content in PM22-inoculated plants increased at 300 mM (45.25%) NaCl, followed by 240 mM (33.42%) and 180 mM (21.42%) NaCl compared with their respective controls (Figure 5c). The salt tolerance index (STI) was markedly decreased in plants with applications of NaCl (Figure 5d). Although a significant increase in STI was noted in plants with applications of salt along with the inoculation of PM22 (Figure 6).

### 2.10. Gene Expression Estimation

**Figure 7:**
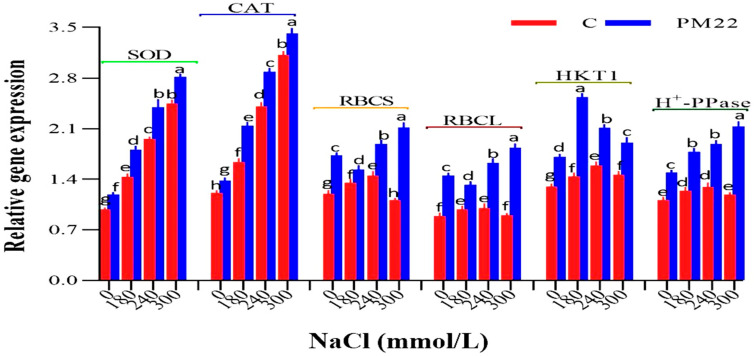
Expression evaluation of genes responsible for antioxidants and other stress tolerance conferring genes in maize with and without the application of *Bacillus safensis* PM22 under different salt stress levels (0, 180, 240, and 300 mM). Soil containing non-inoculated seeds and soil with seeds inoculated with *B. Safensis* (PM22) were the two treatments. The values are the averages of three triplicates with standard errors of the mean. Different alphabets have significantly different values (*p* = 0.05) from each other, according to Tukey’s least significant difference test.

*Bacillus safensis* PM22 was studied for its influence on genes transcription that confers salinity stress tolerance in plants. PM22-inoculated maize plants had greater levels of expression for antioxidant genes (SOD, CAT), photosynthesis responsible genes (RBCS and RBCL), and genes involved in regulating ion balance (HKT1, H^+^-PPase) under salt stress. Moreover, under control conditions, inoculated maize plants exhibited higher expression levels for all the analyzed genes as compared with non-inoculated maize plants (Figure 7).

### 2.11. Amplification of Surfactant Responsible sfp-Gene

*Bacillus safensis* PM22 was tested to analyze whether it possesses a gene for salt stress resistance. The above-mentioned primers produced a distinct band of nearly 675 base pairs (bp) after polymerase chain reaction-mediated amplification of the bio-surfactant-producing sfp gene. The bands were captured on a 2% agarose gel as given in the Supplementary File (Figure S1).

### 2.12. Correlation and Principal Component Analysis of Studied Morpho-Physiochemical Traits and Amplification of sfp Gene

**Figure 8:**
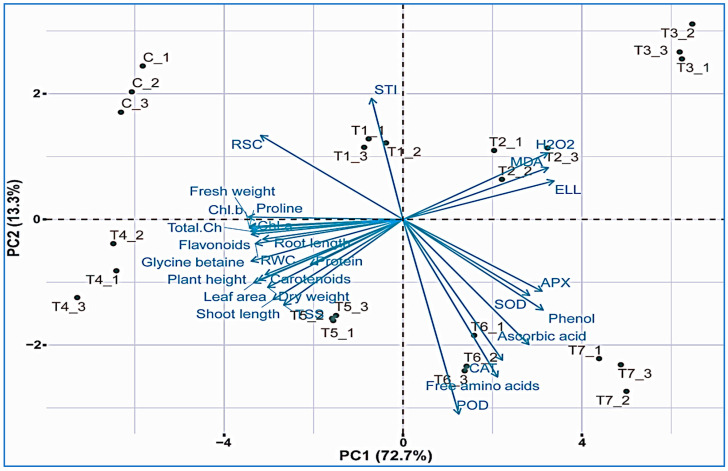
Cluster analysis and PCA biplot analysis between biochemical parameters and antioxidants with plant biomass parameters under various salt increments; shoot length, root length, plant height, fresh weight, dry weight, leaf area, chlorophyll a (Chl a), chlorophyll b (Chl b), total chlorophyll (T. chl), carotenoids, superoxide dismutase (SOD), peroxidases (POD), catalase (CAT), ascorbate peroxidase (APX), ascorbic acid, phenolic content, flavonoid content, relative water content (RWC), electrolyte leakage (ELL), glycine betaine, proline content, protein content, total soluble sugars (TSS), malondialdehyde (MDA), hydrogen peroxide (H_2_O_2_), salt tolerance index (STI), radical scavenging capacity (RSC), and free amino acids.

Correlation analysis was used to summarize the correlation among all the studied parameters of the maize plant. A noteworthy positive correlation was noted among root and shoot length, plant fresh and dry weight, chlorophyll, carotenoids, leaf area, total soluble sugars, total flavonoids, and protein contents. On the contrary, a distinguished negative correlation of root and shoot length, dry and fresh weight, STI, leaf area, and TSS contents was observed with hydrogen peroxide, electrolyte leakage, and malondialdehyde contents. Principle component analysis (PCA) was applied to data to assess the association among all the studied morpho-physiochemical attributes of maize plants. PCA demonstrated a variance of 86% in all parameters. The PCA graph exhibited a negative relationship between antioxidants, osmolytes, sodium contents, organic solutes, radical scavenging capacity, and electrolyte leakage. Although, potassium, pigments and relative water contents, leaf area, and salt tolerance index showed a strong positive correlation with plant growth and biomass (Figure 8).

## 3. Discussion

A high yield and crop production while mitigating abiotic stresses are required for sustainable agriculture. PGPR is well reported for plant growth promotion and stress tolerance [23]. To ensure their survival and prevalence in salinity stress, PGPR has developed extensive physio-chemical processes [24]. The present study has confirmed the efficacy of rhizobacteria in encouraging salt tolerance by endorsing several plant growth-promoting traits. In the present investigation, the bacterial strain indicated high tolerance toward salinity stress. Additionally, it produced significant amounts of IAA, ACC deaminase, siderophore, and EPS under salinity stress. Under saline situations, *Bacillus safensis* PM22 transformed tryptophan to IAA, which is essential for plant growth. PGPR’s synthesis of IAA has also been shown to improve plant growth in previous research, for instance, *Enterobacter* sp., *Bacillus tequilensis* A3, *Bacillus thuringiensis*, *Bacillus* sp., and *Bacillus mycoides* A1 [25,26]. Additionally, it enhances the germination of seeds, root elongation, and improved root hair, all of which aid in crop nutrient uptake [27].

Bacterial strains that produce siderophores are crucial characteristics that help plants grow and develop [28,29]. PGPR promotes the movement of iron in the rhizosphere, making it more available to plants. According to our findings, PM22 was able to generate siderophore under elevated stress conditions. In this regard, the prior study also found that bacteria producing siderophore was found to colonize roots of potato, sugar beet, and radish, resulting in up to 144% increased plant growth in field experiments [30,31]. Several other studies revealed that rhizospheric microorganisms produced siderophores, which increased plant Fe intake and growth characteristics [32,33].

One of several primary ways by which PGPR suppresses ethylene biosynthesis in the plant during abiotic stress conditions is the synthesis of ACC deaminase. In plants, the essential requirement for the generation of the ethylene hormone is ACC [34]. *Bacillus safensis* PM22 produced ACC-deaminase in the current study, which degraded ACC into ammonia and α-ketobutyrate in roots and redirected ethylene synthesis pathways under salt stress. Plant-associated bacteria that produce ACC have been able to withstand abiotic stress by reducing the detrimental impacts of ethylene production [35]. ACC-deaminase-releasing bacteria have been shown in many studies to alleviate the detrimental effects of salinity stress by reducing ethylene production and, as a result, improving plant growth under stress [36,37,38].

In the current study, salinity stress significantly affected the morpho-agronomical prospects of plants, while bacterial inoculation significantly adjusted the affected traits to improve the growth and vigor of plants. In comparison to control plants, the PM22 strain enhanced root and shoot length, plant height, dry and fresh weight, and total leaf area of maize plants. The pigment contents (Chl a, Chl b, total Chl, and carotenoids) were improved by the application of PM22 under salt stress (Figure 2). Previous studies have reported that the augmentation of PGPR encouraged agronomical parameters of wheat in saline soil [39,40]. Kumar et al., 2017 observed increased production of different photosynthetic pigments in PGPR-treated paddy seedlings under salt stress, which confirms the findings of this study [41]. PGPR, *Achromobacter piechaudii*, increased photosynthetic activity in salinity-stressed tomato plants [42]. The synthesis of IAA and ACC-deaminases by PGPR during stress conditions may be responsible for the increased growth of the bacteria-treated plants [43]. EPS synthesis by halotolerant PGPR under salt stress may be responsible for increased chlorophyll content. Under salt stress, exopolysaccharides protected plant seedlings from desiccation [44].

Plants acquire soluble solutes at high salinity to counteract the harmful effects of the saline condition and keep ionic equilibrium in cells [45]. In the current study, PM22 augmented plants under salinity stress improved total soluble sugars and protein content. Plants inoculated with PGPR increased TSS production and protein content under salt stress, according to Kamran et al., 2016 and Sapre et al., 2018 [46,47]. Soluble sugars are osmolytes that help with osmotic changes in abiotic stressors [48]. The activation of stress-related protein production might lead to higher protein content under stress [49].

The RWC of inoculated plants was greater than the control, indicating that they were better acclimated to salty environments. Salt stress reduced root water conductance, causing the flow of water to diminish and stomatal closure to occur [50]. The lowering of RWC in plants reduced photosynthesis and transpiration, according to Li et al., 2020 [51]. In our study, maize inoculated with *Bacillus safensis* PM22 showed decreased Na^+^ accumulation. One of the main reasons for enhancing the survival of rice plants under salt stress by decreasing the availability of Na^+^ ions to plants might be due to EPS synthesis by the PM22 strain [52]. Furthermore, PM22 inoculation displayed an increased amount of K^+^ deposition in maize plants which might be another factor in the alleviation of salt stress caused by extreme salinity. Plants are said to withstand high salt levels by omitting Na^+^, tolerating osmotic stress, and adapting tissue Na^+^ levels [53].

In our research, inoculating maize plants with *Bacillus safensis* PM22 significantly boosted the enzymatic antioxidants (SOD, CAT, POD, and APX) under salinity stress. In comparison to control plants, these antioxidant compounds reduced H_2_O_2_ levels and oxidative damage in PM22-infected plants. The antioxidant properties of PGPR were increased, according to Narayanasamy et al., 2020 [54]. The inoculation of PGPR, on the other hand, reduced the APX level at all salinity dosages administered. In addition, Habib et al., 2016 found that 5-aminolevulinic acid-producing bacteria decreased H_2_O_2_ production and boosted POD, SOD, and CAT antioxidant activity in salt-stressed rice plants [55]. The increased antioxidant enzymatic activities in maize plants revealed that PGPR stimulated antioxidant defense mechanisms, removed harmful free radicals, and improved salt tolerance [56].

Under salt stress, osmoprotectants such as free proline, glycine betaine, and amino acid are formed [57]. These substances control water holding capacity and help protect plants from osmotic stress [9]. Increased proline under salinity stress plays a key role in the osmoregulatory function of plants [58]. The current work reported that PGPR (PM22)-inoculated maize plants showed increased proline and glycine betaine contents in contrast to non-inoculated plants. The increased proline and betaine serve as compatible active solute that maintains membrane stability through osmotic adjustment [59]. The rise in amino acid levels was noted in both non-inoculated and inoculated maize plants with the application of salt stress. Amino acids are the originators or intermediates of metabolites that are crucial for stress tolerance in both biotic and abiotic stresses [60]. This indicates their function in reinforcing host plant defenses via a variety of methods, including increased protein synthesis, higher growth rate under abiotic stressors, relief of biotic stress via delayed cell death, and pathogenic invasion avoidance in host plant tissues [61].

In the present study, radicals scavenging capacity, electrolyte leakage (EL), and malondialdehyde (MDA) contents were markedly reduced in *Bacillus safensis* PM22-inoculated maize plants, contrary to DPPH scavenging capacity. Salt stress substantially enhanced the MDA concentration and EL in the current investigation. EL and MDA are essential physiological markers for assessing the integrity and cell membrane permeability, which are both altered by abiotic stress. One of the primary consequences of salt is an excess of reactive oxygen species (ROS) in plant cells, which causes peroxidation of lipids in the membrane and thus damages the cell membrane, raising EL percentage and leaf MDA contents [62]. In comparison to non-inoculated plants, inoculated plants exhibit reduced EL and MDA content at all NaCl doses. Plant-beneficial rhizospheric microbes (PBRM) inhibit oxidative damage which causes to enhances the stability of plant cells [63,64]. Tyagi et al., 2017 discovered that PBRM-induced phenol-soluble modulins contribute to free radical elimination, resulting in the membrane and cell wall strengthening via decreased protein and lipid oxidation [65]. PM22 may modulate membrane stability by scavenging excess radicals generated within the plant cells. In terms of H_2_O_2_ and MDA, our results are similar to those of Bharti et al., 2014, who found that PGPR-inoculated maize and white clover plants decreased oxidative stress indicators in salinity stress conditions [66].

Moreover, in the current investigation, *Bacillus safensis* PM22 stimulated flavonoid, phenolic, and ascorbic acid synthesis mechanisms in maize exposed to salinity stress and enhanced plant tolerance to salinity. Bistgani et al., 2019, demonstrated that by inoculation with PGPR in pea plants, phenolic content was accumulated to mitigate salt stress [67]. While in salt stress, the large deposition of flavonoids and phenolic content in ST-PGPR-treated plants may aid in the elimination of ROS and decomposition of H_2_O_2_ to avoid oxidative damage [68,69]. Previous studies have shown that increased concentration of ascorbic acid reduced membrane damage and improved chlorophyll content in *Solanum lycopersicum* [70,71]. Previous research has also demonstrated that ascorbic acid expression improves salt tolerance in plant cells, resulting in increased plant growth [72]. The salt tolerance index (STI) was considerably increased by PM22 compared with non-inoculated salt-stressed maize plants.

The *sfp* gene, which creates bio-surfactants, aids in the improvement of PGPR and plant root interface resulting in improved root colonization and plant yield [73]. Additionally, bio-surfactant may have assisted bacterial cells in soil by enhancing their ability to create compounds containing essential metal ions and phytonutrients [74]. Moreover, the effective penetrating activity, gelling, wetting, and bifunctional characteristics of these biosurfactants render them an excellent dissipating agent, this might help plant growth-promoting rhizobacteria colonize plant roots and make plant hormones and siderophores more available to the plant [75].

## 4. Materials and Methods

### 4.1. Bacterial Strain Assessment

The previously isolated bacterial strain, *Bacillus safensis* PM22, was acquired from Plant-Microbe Interactions Lab., operating in Quaid-i-Azam University, Islamabad 45320, Pakistan. The strain was assessed for its salt lenience potential against various concentrations (0.0, 0.5, 1.0, and 1.5 mol/L) of sodium chloride (NaCl) in the Luria–Bertani (LB) broth medium. The inoculated medium was then subjected to continuous shaking for 72 h at 32 ± 2 °C in a shaking incubator. Following that, the optical density (OD) of samples was recorded at 600 nm to assess bacterial growth at various salt concentrations.

### 4.2. Quantification of Plant Growth-Promoting Traits of the Bacterium under Salt Stress

Indole-3-Acetic Acid (IAA) was determined by means of the method of Khan et al., 2016 [76]. *B. Safensis* PM22 was inoculated in 20 mL of LB broth (L-Tryptophane 1 mg/mL) having different salt concentrations (0.0, 0.25, 0.5, and 0.75 mol/L) and kept at 30 ± 2 °C in a shaking incubator (200 rpm) for 7 days. The PM22 cultures were centrifuged at 10,000× *g* for 10 min at 4 °C. One milliliter of supernatant was mixed with 2 mL of Salkowski reagent (7.9 M of sulfuric acid (H_2_SO_4_), 12 g of ferric chloride (FeCl_3_) per liter) and reserved in the dark for 30 min. The final obtained sample (reddish color) was measured at 535 nm on a spectrophotometer after 30 min. The quantity of IAA was found by means of the standard curve of absolute IAA that was established independently.

The siderophore was measured using PM22 cultures grown with and without NaCl by following the protocol mentioned in Hu and Xu, 2011 [77]. Briefly, 1 mL LB broth media was added to 1.5 mL sterilized tubes, and 10 μL of fresh culture (108 CFU/mL) was added, respectively. The tubes were kept in an incubator at 30 °C for 48 h. Afterwards, the tubes were centrifugated for 10 min at 16,800× *g* and 0.5 mL of supernatant was allowed to mix with 0.5 mL of Chrome azurol sulfonate (CAS) reagent. In a spectrophotometer, the optical density was measured at 630 nm after 20 min. Siderophore production was calculated using the formula given below:

$$\mathrm{PSU}=\left(\mathrm{Ar}-\mathrm{As}/\mathrm{Ar}\right)\times 100$$

where As is the absorbance of the inoculated sample and Ar is the reference (CAS + NaCl concentration + non-inoculated broth).

Exopolysaccharides were determined quantitatively by utilizing the procedure given by Zainab et al., 2020 [78]. *B. safensis* PM22 was inoculated and grown in 50 mL of ATCC No. 14 media with various salt concentrations (0.0, 0.25, 0.50, and 0.75 mol/L) and was kept in an incubator for 24–48 h at 32 ± 2 °C and 150× *g*. Subsequently, 72-h old bacterial cultures were centrifugated at 10,000× *g* for 20 min. The obtained pellets were treated with two volumes of acetone and kept at 40 °C for about 12 h. The pellet was then dried at 100 °C, weighed and exopolysaccharide (EPS) was determined in mg/mL of the dry mass.

The ACC deaminase quantification assessment was carried out on late log phase bacterial cultures. The washed (0.1 M Tris HCl) pellets were added to 3 mM ACC enriched DF medium with and without salt stress. The cultures were then kept in an incubator for 72 h. The quantity of ACC was determined by matching the OD (540 nm) of samples with the α-ketobutyrate standard curve drawn in the range 10–200 μmol (Afridi et al., 2019) [79].

### 4.3. Experimental Setup

The soil used in the current study was obtained from the field of Quaid-i-Azam University, Islamabad, Pakistan (33.7470° N, 73.1371° E). The upper surface of the soil was removed and 6–12 inches below the surface, soil was collected, air-dried, crumpled, sieved (2 mm sieve), and was twice autoclaved at 121 °C for 40–60 min [80]. Maize seeds (CZP-13001) were provided by National Agricultural Research Center (NARC). Maize seeds were surface sterilized by using 70% ethyl alcohol (C_2_H_5_OH) for 5 min and then 0.1% mercuric chloride (HgCl_2_) for about 1 min and then rinsed three to four times using autoclaved distilled water. The 48 h-old LB-broth culture of *Bacillus safensis* PM22 was spun in a centrifuge for 10 min at 10,000× *g* and the pellets were rinsed with NaCl (0.85%) solution and immersed in sterilized water to keep at 107 CFU/mL (O.D 0.5). Maize seeds were kept in a solution containing PM22 for 3–4 h, whereas nonprime maize seeds were immersed in decontaminated water reserved as control [81]. Six seeds were sown in each plastic pot holding 200 g of autoclaved soil (EC 3.6 dS/m, pH 7.2, 0.14% organic carbon, 0.29% organic matter, 91 kg/hectare accessible nitrogen). The level of salinity in the pots was sustained at 0, 180, 240, and 300 mM. Two treatments (non-inoculated and PM22-inoculated seeds) were planned in a complete randomized design and pots were situated in the growth chamber. Salinity was imposed on plants on the approaching 12th day of germinated maize plants. 20 mM NaCl doses were applied once a day to the respective plants till the accomplishment of 180, 240, and 300 mmol final concentrations. The (EC) electrical conductivity of leached water from the pots through openings in the bottom was equivalent to the EC of irrigated saline solution, confirming the desired salt stress. The humidity in the growth chamber was maintained at 60–80%, the light period day-night was 12 h, and the temperature ranges were 20 °C and 32 °C for the night and day, respectively. After 28 days of germination, the plants were collected. Roots were washed with tap water to get rid of debris and soil. The pots experiment was designed as; control; noninoculated pots with no (0 mM) or applied concentrations of salts (180, 240, and 300 mM) and PM22; pots treated with PM22 with no (0 mM) and different applications of salinity stress (180, 240, and 300 mM).

### 4.4. Determination of Morphological and Physicochemical Parameters of Maize

After harvesting, the root and shoot length were measured by a standard measuring scale. For fresh weight (FW) and dry weight (DW), different plant parts were weighed before and after incubation in an oven (105 °C), respectively. The leaf surface area was calculated using the equation;

$$\mathrm{Leaf}\;\mathrm{area}\;\left(\mathrm{LA}\right)=\left(L\right)\;\left(W\right)\;\left(0.75\right)$$

Leaf area was given in cm^2^ per plant, Where, L = leaf length, and W = leaf width (Ahmad et al., 2021) [82].

### 4.5. Pigments Content

Post-harvested fresh leaves were used to measure pigment contents. The plant tissues were smashed to a fine powder and placed in 80% acetone (*v*/*v*). The contents of chlorophyll a, b (Chl a, Chl b), total chlorophyll, and carotenoids were calculated (using formula given below) after reading the supernatant at absorbance 663, 647, and 470 nm [83].

$$\mathrm{Chlorophyll}\;a\;\left(\mathrm{Chl}\;a\right)=\left(12.7\times \;A\;663\right)\;-\;\left(2.49\times \;A\;645\right)\;\mathrm{Chlorophyll}\;b\;\left(\mathrm{Chl}\;b\right)=\left(12.9\times \;A\;645\right)\;-\;\left(4.7\times \;A\;663\right)\;\mathrm{Total}\;\mathrm{Chlorophyll}\;=\mathrm{Chlorophyll}\;a\;+\;\mathrm{Chlorophyll}\;b\;\mathrm{Carotenoids}=4\times \mathrm{OD}$$

### 4.6. Total Soluble Sugars and Protein Contents in Leaf

The techniques of He et al., 2019 were used to determine total soluble sugars (TSS) and protein content of maize leaves [59]. Briefly, 500 mg of fresh tissues immersed in water were kept at 100 °C for half an hour. All extracts from their respective samples were permitted to mix with sulfuric acid and phenol. The absorbance (OD, 485 nm) was recorded and presented in micrograms of TSS in a gram of fresh weight of plant (mg/g FW). The contents of protein were estimated by homogenizing 0.15 g fresh shoots at 4 °C in phosphate buffer (0.5 M) of pH 6.8 and were centrifuged at 10,275× *g* (4 °C) for half an hour. The OD of samples was recorded at 650 nm. The total quantity of protein was estimated by means of the standard curve for the bovine serum albumin (BSA) and presented as mg/g FW.

### 4.7. Relative Water Content (RWC) and K^+^ and Na^+^ Analysis

For each treatment, 0.5 gm of the leaf was used for RWC tests. The leaves were kept in dH_2_O for 4 h. The turgid weight (TW) was determined after the samples were rapidly wiped dry. The oven-dried (80 °C for 24 h) samples were used to determine the dry weight (DW) of the samples [84]. RWC was estimated using the following formula:

$$\mathrm{RWC}\;\left(\%\right)=\frac{\left(\mathrm{FW}\;-\;\mathrm{DW}\right)}{\left(\mathrm{TW}\;-\;\mathrm{DW}\right)}\times 100$$

Dry maize plant materials were wet digested with HClO_4_-HNO_3_ (1:3) and K^+^ and Na^+^ were measured using a flame photometer (Systronics-130, Ahmedabad, India). Plant materials (dried samples) were digested with a digestion mixture (10 mL) and were stored overnight. The sample was heated on a hot plate until the appearance of white color. The samples were diluted with distilled water (50 mL) and were filtered by passing through Whatman filter paper (No. 42). The filtrates were used to determine K^+^ and Na^+^ concentrations [84].

### 4.8. Antioxidant Activities in Maize Leaves

The changes in the concentration of defense enzymes and their activities in *Zea mays* L. plants under different treatments were estimated. The homogenized extract was prepared to take 500 mg maize leaves in 5000 µL phosphate buffer (50 mmol) having a pH of 7.8. The extracts were centrifugated at 12,000× *g* for 20 min at 4 °C, with the supernatant used to test antioxidative enzyme activity. The activities of ascorbate peroxidase (APX), catalase (CAT), superoxide dismutase (SOD), and peroxidase (POD) were evaluated by utilizing the technique of Zhang et al., 2018 [85].

### 4.9. Proline, Glycine Betaine, and Free Amino Acids Substances

The proline estimation was executed using the ninhydrin colorimetric method of Du et al., 2019 [86]. Plant leaf tissue (0.5 g) was ground in 3% aqueous sulfosalicylic acid (5 mL), boiled for 10 min, and were filtered out. Acid-ninhydrin, glacial acetic acid, and the filtrate were mixed with 2:2:2 in a test tube and were kept in a water bath at 100 °C for 30 min. Then, toluene (4 mL) was introduced to the extract and the absorbance was noted (520 nm). The content of proline was determined using the L-proline standard curve and represented in micrograms proline per gram fresh weight of plant (µg/g FW).

The quantity of glycine betaine (GB) was quantitatively estimated using the Reinecke salt-dependent colorimetric method [83]. Leaf samples (500 mg) were ground and extracted in a mixture of methanol, chloroform, and water (6:2.5:1.5). The mixtures were spun at 10,280× *g* for 10 min after the samples were boiled at 40 °C for 10 min. Then, 3 mL of extract containing the absolute solution of Reinecke’s salt (5 mL), was allowed to react for 6 h at 4 °C. The supernatant was condensed with ethyl ether (3 mL), after centrifuging. The precipitate was re-suspended in 3 mL acetone (70%) and ODs were recorded at 525 nm. The standard curve of GB was created using standard samples GB and a unit of mg/g FW.

The total amino acid content of plant tissues was assessed by the technique previously designated by Alexander et al., 2021 [87]. The 100 mg plant leaf samples were homogenized in 80% ethanol, and the extract was treated with citrate buffer (0.2 M) of pH 5 and ninhydrin reagent in an equal volume (1% ninhydrin). The reaction mixture was incubated at 95 °C for 15 min. The absorbance was measured at 570 nm after the samples were centrifuged and cooled to room temperature.

### 4.10. Radical Scavenging Capacity of Maize

The 2,2-diphenyl-1-picrylhydrazyl (DPPH) radical scavenging activity of extracts was estimated from the whitening of purple-colored DPPH solution [88]. The 20 μL samples suspended in methanol were added with 0.1 mM DPPH solution in 180 μL volume. After 30 min, bleaching the color of the mixtures was scored at 517 nm. Inhibition of DPPH was assessed in percent using the following equation:

$$I\;\left(\%\right)=\left(\mathrm{Ac}-\mathrm{As}/\mathrm{Ac}\right)\times 100$$

where the absorbance of the control reaction is denoted by Ac (reagents without sample), and the absorbance of the extracts is denoted by As.

### 4.11. Determination of Oxidative Stress by Electrolyte Leakage, Malondialdehyde, and Hydrogen Peroxide Contents

Electrolyte leakage (EL) was estimated according to the method used by Asghari et al., 2020 [89]. The pieces of leaf were immersed in double distilled water at normal temperature for 24 h. After determining EC1 (electrical conductivity of the solution), the samples were kept at 95 °C for 20 min, and EC2 (electrical conductivity of the incubated solution) was recorded after cooling. EL was measured by the formula given below:

$$\mathrm{EL}\;\left(\%\right)=\left(\mathrm{EC}1/\mathrm{EC}2\right)\times 100$$

The concentration of hydrogen peroxide (H_2_O_2_) was determined using an assay outlined by Islam et al., 2016 [90]. 0.5 g fresh leaf tissues were dissolved in 5 mL trichloroacetic acid (0.1% *w*/*v*) and the sample was spun (12,000× *g*) for 15 min. Ten mM potassium phosphate buffer (pH 7.0) and 1M potassium iodide were mixed in 1:1 ratio with the supernatant, The OD of the sample was measured at 390 nm after the samples were vortexed. The concentrations of H_2_O_2_ were estimated using a standard curve with known H_2_O_2_ values.

The 2-thiobarbituric acid (TBA) responsive metabolites, primarily malondialdehyde (MDA) were used to measure lipid peroxidation in plant tissues [47]. Concisely, 0.5 g of leaf tissue were saturated in ethanol (80%) and 1 mL extract and TBA were mixed (Trichloroacetic acid, 0.5% TBA in 20% in *w*/*v*). For control, 1 mL extract was introduced to 1 mL (20 %) trichloroacetic acid. The mixture was boiled at 90 °C for 30 min and then cooled down to normal room temperature and O-D was recorded at 440, 532, and 600 nm.

### 4.12. Total Flavonoids and Phenol Estimation

The aluminum chloride method was used to measure flavonoid content [91]. In brief, 0.5 mL of extract was treated with 0.1 mL of potassium acetate (1 M), 1.5 mL of methanol, 0.1 mL of aluminum chloride, and 8.2 mL of distilled water, and the absorbance (O.D) of the sample was noted at 415 nm following incubation in darkness for half an hour at normal temperature. To analyze the flavonoid content of samples, the standard curve was set with various quercetin concentrations. Total flavonoid content was presented in terms of mg/g quercetin per gram fresh weight of plant (mg/g FW).

To estimate total phenol contents, the procedure of Forouzi et al., 2020 and Ahmad et al., 2001 was followed [92,93]. Briefly, leaf alcoholic extract (20 μL) was mixed with deionized water (1.15 mL) and folin ciocalteu reagent (0.1 mL). Then, 1M sodium carbonate (300 μL) was added to the solution after 5 min and the sample was stored in shade at room temperature for 20 min. Assessment of total phenol was accomplished at 760 nm. Various concentrations of gallic acid were employed for the depiction of the standard curve in triplicates. The total amount of phenol content was presented in mg gallic acid (GA) per gram of fresh weight.

### 4.13. Measurement of Ascorbic Acid Contents and Salt Tolerance Index

Ascorbic acid contents were measured according to the protocol used by Kartik et al., 2020 [94]. Leaf samples (1 g) were made homogenous in 5% ice-cold trichloroacetic acid (TCA). The sample was spun (10,000× *g*) for 10 min at 4 °C. Afterward, the supernatant (0.5 mL) was combined with 200 µL of sodium molybdate (0.66%), 200 µL H_2_SO_4_ (0.05 N), 0.1 mL sodium phosphate (0.025 mM), and the mixture was kept in a water bath at 60 °C for 40 min. Then, the samples were cooled using water and spun at 4000× *g* for 5–6 min. The final supernatant was obtained and OD was recorded at 660 nm using a spectrophotometer. The ascorbic acid contents were represented in µg/g fresh weight.

Saghafi et al., 2019 was followed for the determination of salt tolerance indices (STI) of halotolerant bacteria augmented and non-augmented plants as below [95]:

STI=DWS or DWH / DWC

where DWS is the dry weight of salt-stressed plants, DWH is PM22-inoculated plants under salinity stress and DWC is the dry weight of maize plants without salinity stress and PM22 inoculation.

### 4.14. Transcription Analysis of salt Stress Tolerance Responsible Genes

The Qiagen RNeasy Plant Mini kit was used to extract total RNA from control, salt-stressed, and *B. safensis*-inoculated maize plants [96]. The expression of six genes involved in establishing salinity tolerance in maize was measured using quantitative real-time PCR. The transcript levels of superoxide dismutase (SOD), catalase (CAT), RBCS (encoding Rubisco small subunit), RBCL (Rubisco large subunit), H^+^-PPase (encoding Proton (H^+^)-pumping pyrophosphatase), and HKT1 (encoding high-affinity K^+^ transporter 1) were measured. The 2^−ΔΔCt^ technique was used to measure the expression level of TUB (encoding -tubulin), which was used as an internal reference gene. Three replicates were collected from each treatment, and gene-specific forward and reverse primers were employed (see Table S1 in the Supplementary File) [97,98]. The protocol of Livak and Schmittgen, 2001 was used for the preparation of the PCR mixture [99]. Kamal et al., 2021 described PCR amplification protocol was used [100].

### 4.15. Genomic DNA Extraction, Amplification of sfp Gene

*Bacillus safensis* PM22 genomic DNA was isolated using the phenol-chloroform technique of Ali et al., 2021 [81]. The isolated DNA was utilized as a template for biosurfactant RNA gene (*sfp*) amplification (cloning) through PCR, by oligonucleotide *sfp* R: 5′-TTATAAAAGCTCTTCGTACG-3′ and *sfp* F: 5′-ATGAAGATTTACGGAATTTA-3′ primers. Amplification (PCR) was executed with a DNA Thermal Cycler of PTC-200, MJ Research, Waltham, MA, USA. PCR was comprised of the Step-Cycle program set at 94 °C for 1 min (denaturing), at 46 °C for 30 s (annealing), and polymerization at 72 °C for 1 min, for an overall of 25 cycles. After amplification, the PCR product was examined on 2% agarose gel by gel electrophoresis. In the Gel Doc system, the predicted DNA band of the *sfp* gene (675 bp) was detected [101].

### 4.16. Statistical Analysis

All the data were collected in triplicates for each treatment. We determined the mean values and standard errors. Using Statistix version 8.1, analysis of variance (ANOVA) was used to examine the data, and pairwise comparisons between treatment means were performed using the LSD test at *p* = 0.05. GraphPad Prism version 8.0.1 was used to visualize data in the form of graphs. Principal Component Analysis (PCA) was analyzed by using Xlstat version 2022.1.1 software.

## 5. Conclusions

*Bacillus safensis* PM22 showed significantly high (1.5 mmol/L) salt tolerance based on that which is considered a halotolerant specie. Further, PM22 activities such as IAA, ACC-deaminase, Siderophore, and EPS under salinity stress revealed it as a PGPR. This promising strain has a great potential in the growth promotion of maize plants under salinity stress by induction of photosynthetic efficacy, deposition of soluble solutes, level of non-enzymatic and enzymatic antioxidants, osmoprotectants production, and reduction in oxidative stress markers. Genetic characterization of PM22 also supported its roles in plant growth promotion and multi-stress tolerance. PM22 can be used as a stress-tolerant PGPR in the soil to face the increasing challenges of salinity. Therefore, the use of PGPR with the potential to induce salinity tolerance could be a useful strategy in sustainable agriculture. Considering the above-mentioned findings, PM22 can be used as a bio-inoculant or bio-fertilizer to improve plant growth, yield, and salt stress tolerance.

## Figures and Tables

**Table 1:** Impacts of various treatments on morphological traits of PM22-inoculated maize at different salt stress levels (0, 180, 240, and 300 mM). Soil containing non-inoculated and soil with seeds inoculated with *B. Safensis* (PM22) were the two treatments. The values are the averages of three triplicates with standard errors of the mean. Lowercase letters indicate significantly different values (*p* = 0.05) from each other, according to Tukey’s least significant difference test.

Treatments	Shoot Length (cm)	Root Length (cm)	Plant Height (cm)	Fresh Weight (gm)	Dry Weight (gm)	Leaf Area (cm^2^)
0 − PM22	28.63 ± 1.23 bcd	17.76 ± 0.78 b	50.43 ± 1.97 b	7.93 ± 0.21 b	0.66 ± 0.01 b	22.36 ± 0.23 b
0 + PM22	33.21 ± 1.89 a	23.83 ± 1.12 a	63.06 ± 3.34 a	11.66 ± 0.3 a	0.87 ± 0.02 a	28.62 ± 0.88 a
180 − PM22	24.66 ± 0.91 cde	14.00 ± 0.84 cd	41.16 ± 1.75 cd	5.55 ± 0.19 c	0.49 ± 0.01 e	18.71 ± 0.64 c
180 + PM22	31.13 ± 2.05 b	17.30 ± 0.65 b	48.49 ± 2.76 b	7.28 ± 0.16 b	0.63 ± 0.01 bc	20.72 ± 0.33 b
240 − PM22	21.00 ± 0.74 e	12.81 ± 0.65 d	36.03 ± 1.62 d	3.92 ± 0.11 de	0.42 ± 0.00 f	12.53 ± 0.43 d
240 + PM22	28.80 ± 1.43 bc	16.33 ± 0.74 bc	45.21 ± 2.13 bc	5.03 ± 0.12 cd	0.57 ± 0.01 cd	17.39 ± 0.39 c
300 − PM22	16.31 ± 0.81 f	10.01 ± 0.71 e	28.34 ± 1.48 e	2.93 ± 0.14 e	0.35 ± 0.01 g	11.01 ± 0.56 e
300 + PM22	21.16 ± 1.58 e	13.70 ± 0.49 d	34.86 ± 2.17 d	4.62 ± 0.09 cd	0.55 ± 0.02 d	14.47 ± 0.52 d

## Data Availability

The paper reflects the authors’ own research and analysis in a truthful and complete manner. The paper is not currently being considered for publication elsewhere. All authors have been personally and actively involved in substantial work leading to the paper and will take public responsibility for its content.

## Supplementary Materials

The following supporting information can be downloaded at: https://www.mdpi.com/article/10.3390/plants11131721/s1, Figure S1: PCR amplified product of the sfp gene; Table S1: DNA primers used for quantitative reverse transcription polymerase chain reaction (qRT-PCR).
