# Supplementary material for: Biochemical Characterization of Halotolerant Bacillus safensis PM22 and Its Potential to Enhance Growth of Maize under Salinity Stress
Source: Plants (Basel). 2022 Jun 29;11(13):1721. doi: 10.3390/plants11131721 (PMC9268828; doi:10.3390/plants11131721)
Supplement: Supplementary file 1 [file plants-11-01721-s001.zip › plants-1773336-supplementary.pdf]

Table S1. DNA primers used for quantitative reverse transcription polymerase chain reaction. (qRT-PCR)

Gene	Primer	Sequence	Reference
SOD	Forward	5′-TGGAGCACCAGAAGATGA-3′	Vwioko et al, 2017
	Reverse	5′-CTCGTGTCC ACCCTTTCC-3′	
CAT	Forward	5′-CTGAACGTGTTGTGCATGCA-3′	
	Reverse	5′-TAGATCCTTCGTCGCATGGC-3′	
RBCS	Forward	5′-AAGGTCGGCTTCGTGTA-3′	Chen et al, 2016
	Reverse	5′-CTGCGTCTGCTTGATGT-3′	
RBCL	Forward	5′-TGCTCGTCGTTCTCCAGTGTAT-3′	
	Reverse	5′-GCTGCCGTTGAGGAAGGTATTG-3′	
HKT1	Forward	5′-TCTTCATCGTCGTCATCTG-3′	
	Reverse	5′-CCTTCCACACTCCACTTG-3′	
H^+-PPase	Forward	5′-GGTATTCAGTGGTGTGCTAT-3′	
	Reverse	5′-GGTGGTCCTTCGTCCTTA-3′	

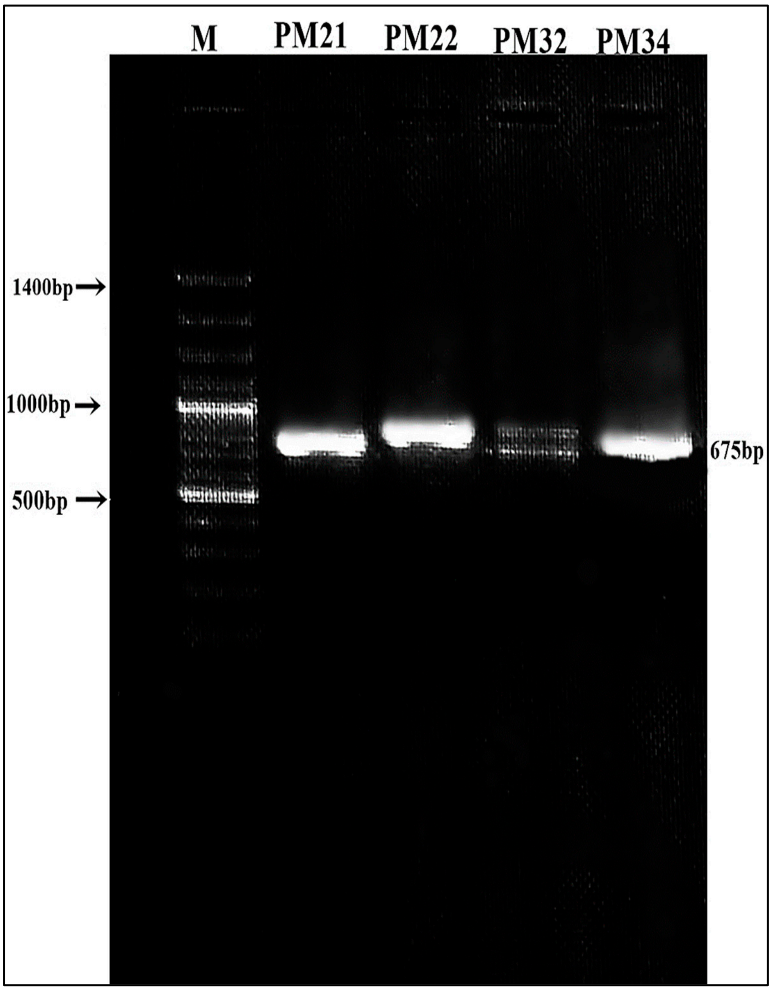

Figure S1. PCR amplified product of the *sfp* gene
